# Supplementary material for: A Likelihood Approach for Real-Time Calibration of Stochastic Compartmental Epidemic Models
Source: PLoS Comput Biol. 2017 Jan 17;13(1):e1005257. doi: 10.1371/journal.pcbi.1005257 (PMC5240920; doi:10.1371/journal.pcbi.1005257)
Supplement: S1 File — (TAR.GZ) [file pcbi.1005257.s014.tar.gz › HSPH_Online-SI-Revision/Additions/MSS11/n100-extreme-SEITR/n100-extreme-SEITR_NEW2_table8.pdf]

1	newly 3 weeks spec	108.	190.79	{176., 205.}	76.6574	0.246277	newly 3 weeks cumul	716.	860.53	{838., 881.}	20.1858	0.0797724	newly 5 weeks spec	52.	90.6	{78., 101.}	74.2308	0.238923	newly 5 weeks cumul	854.	1080.88	{1057., 1111.}	26.5667	0.102249	newly 43 weeks cumul	939.	1283.32	{1255., 1316.}	36.6688	0.135598
2	newly 3 weeks spec	102.	0.07	{0., 0.}	99.9314	2.9386	newly 3 weeks cumul	593.	0.97	{1., 1.}	99.8364	2.82402	newly 5 weeks spec	36.	0.02	{0., 0.}	99.9444	2.5363	newly 5 weeks cumul	673.	1.05	{1., 1.}	99.844	2.81296	newly 50 weeks cumul	712.	1.05	{1., 1.}	99.8525	2.83743
3	newly 3 weeks spec	169.	0.11	{0., 1.}	99.9349	3.11789	newly 3 weeks cumul	920.	0.98	{1., 1.}	99.8935	3.03397	newly 5 weeks spec	52.	0.03	{0., 0.}	99.9423	2.686	newly 5 weeks cumul	1072.	1.09	{1., 1.}	99.8983	3.01259	newly 52 weeks cumul	1137.	1.1	{1., 1.}	99.9033	3.02816
4	newly 3 weeks spec	137.	14.87	{10., 19.}	89.146	0.979649	newly 3 weeks cumul	790.	83.38	{74., 92.}	89.4456	0.97811	newly 5 weeks spec	41.	5.18	{2., 8.}	87.3659	0.952844	newly 5 weeks cumul	909.	97.68	{88., 107.}	89.2541	0.969816	newly 50 weeks cumul	985.	105.08	{96., 115.}	89.332	0.972856
5	newly 3 weeks spec	34.	0.	{0., 0.}	100.	2.53148	newly 3 weeks cumul	171.	1.04	{1., 1.}	99.3918	2.22095	newly 5 weeks spec	11.	0.	{0., 0.}	100.	2.04139	newly 5 weeks cumul	195.	1.04	{1., 1.}	99.4667	2.27799	newly 52 weeks cumul	201.	1.04	{1., 1.}	99.4826	2.29115
6	newly 3 weeks spec	5.	0.12	{0., 1.}	97.6	1.58596	newly 3 weeks cumul	60.	1.22	{1., 2.}	97.9667	1.74238	newly 5 weeks spec	2.	0.05	{0., 0.}	97.5	1.25802	newly 5 weeks cumul	63.	1.34	{1., 2.}	97.873	1.71079	newly 51 weeks cumul	64.	1.34	{1., 2.}	97.9063	1.71763
7	newly 3 weeks spec	469.	11.78	{7., 16.}	97.4883	1.62192	newly 3 weeks cumul	2142.	50.59	{41., 61.}	97.6382	1.63176	newly 5 weeks spec	221.	5.87	{3., 9.}	97.3439	1.61449	newly 5 weeks cumul	2717.	65.19	{53., 76.}	97.6007	1.62463	newly 43 weeks cumul	3226.	81.43	{69., 95.}	97.4758	1.60165
8	newly 3 weeks spec	225.	0.19	{0., 1.}	99.9156	3.16917	newly 3 weeks cumul	1088.	3.11	{1., 5.}	99.7142	2.61257	newly 5 weeks spec	80.	0.01	{0., 0.}	99.9875	2.89309	newly 5 weeks cumul	1321.	3.19	{1., 6.}	99.7585	2.68764	newly 50 weeks cumul	1472.	3.22	{1., 6.}	99.7813	2.73267
9	newly 3 weeks spec	19.	0.06	{0., 0.}	99.6842	2.21875	newly 3 weeks cumul	167.	1.02	{1., 1.}	99.3892	2.23068	newly 5 weeks spec	5.	0.	{0., 0.}	100.	1.69897	newly 5 weeks cumul	179.	1.04	{1., 1.}	99.419	2.24081	newly 52 weeks cumul	188.	1.04	{1., 1.}	99.4468	2.26212
10	newly 3 weeks spec	613.	93.68	{82., 104.}	84.7178	0.818338	newly 3 weeks cumul	2661.	446.82	{428., 466.}	83.2086	0.775155	newly 5 weeks spec	260.	42.43	{34., 50.}	83.6808	0.791745	newly 5 weeks cumul	3324.	552.58	{531., 571.}	83.3761	0.779431	newly 48 weeks cumul	3949.	634.08	{612., 657.}	83.9433	0.794495
11	newly 3 weeks spec	448.	1.19	{0., 3.}	99.7344	2.88257	newly 3 weeks cumul	2137.	6.7	{1., 14.}	99.6865	2.63522	newly 5 weeks spec	237.	0.54	{0., 2.}	99.7722	2.92811	newly 5 weeks cumul	2689.	7.97	{2., 15.}	99.7036	2.63679	newly 47 weeks cumul	3221.	8.72	{2., 15.}	99.7293	2.67046
12	newly 3 weeks spec	209.	0.56	{0., 2.}	99.7321	2.88527	newly 3 weeks cumul	1086.	3.06	{1., 6.}	99.7182	2.63574	newly 5 weeks spec	68.	0.05	{0., 0.}	99.9265	2.78251	newly 5 weeks cumul	1294.	3.5	{1., 7.}	99.7295	2.65519	newly 48 weeks cumul	1442.	3.64	{1., 7.}	99.7476	2.67519
13	newly 3 weeks spec	300.	0.88	{0., 2.}	99.7067	2.83686	newly 3 weeks cumul	1508.	5.8	{2., 10.}	99.6154	2.49117	newly 5 weeks spec	131.	0.23	{0., 1.}	99.8244	2.90824	newly 5 weeks cumul	1818.	6.4	{2., 10.}	99.648	2.5279	newly 49 weeks cumul	2109.	6.55	{2., 11.}	99.6894	2.585
14	newly 3 weeks spec	681.	225.2	{205., 245.}	66.931	0.481507	newly 3 weeks cumul	2981.	914.72	{886., 939.}	69.315	0.513193	newly 5 weeks spec	331.	125.74	{111., 138.}	62.0121	0.421826	newly 5 weeks cumul	3718.	1209.26	{1183., 1236.}	67.4755	0.487853	newly 42 weeks cumul	4474.	1583.68	{1552., 1615.}	64.6026	0.451088
15	newly 3 weeks spec	238.	0.93	{0., 2.}	99.6092	2.70501	newly 3 weeks cumul	1233.	3.9	{1., 7.}	99.6837	2.63263	newly 5 weeks spec	100.	0.43	{0., 2.}	99.57	2.67859	newly 5 weeks cumul	1479.	5.03	{2., 8.}	99.6599	2.5713	newly 43 weeks cumul	1667.	6.18	{2., 11.}	99.6293	2.51892
16	newly 3 weeks spec	233.	220.41	{204., 239.}	6.74249	0.0306685	newly 3 weeks cumul	1269.	941.07	{913., 961.}	25.8416	0.129933	newly 5 weeks spec	91.	116.14	{102., 126.}	27.7143	0.104713	newly 5 weeks cumul	1497.	1217.72	{1185., 1247.}	18.656	0.0897456	newly 46 weeks cumul	1647.	1523.29	{1482., 1554.}	7.51123	0.0339871
17	newly 3 weeks spec	1.	67.89	{58., 79.}	6689.	1.82831	newly 3 weeks cumul	62.	442.12	{426., 457.}	613.097	0.852986	newly 5 weeks spec	0.1	19.27	{12., 27.}	19170.	2.26832	newly 5 weeks cumul	64.	497.06	{477., 518.}	676.656	0.890048	newly 52 weeks cumul	64.	519.75	{498., 542.}	712.109	0.9094
18	newly 3 weeks spec	93.	0.	{0., 0.}	100.	2.96848	newly 3 weeks cumul	605.	1.04	{1., 1.}	99.8281	2.76971	newly 5 weeks spec	53.	0.	{0., 0.}	100.	2.72428	newly 5 weeks cumul	722.	1.04	{1., 1.}	99.856	2.8465	newly 51 weeks cumul	795.	1.04	{1., 1.}	99.8692	2.88833
19	newly 3 weeks spec	465.	1.04	{0., 2.}	99.7763	2.93959	newly 3 weeks cumul	2284.	5.25	{1., 10.}	99.7701	2.78545	newly 5 weeks spec	217.	0.44	{0., 2.}	99.7972	2.99806	newly 5 weeks cumul	2812.	6.43	{1., 12.}	99.7713	2.75817	newly 46 weeks cumul	3336.	7.2	{1., 13.}	99.7842	2.7781
20	newly 3 weeks spec	75.	0.07	{0., 0.}	99.9067	2.80506	newly 3 weeks cumul	401.	0.99	{1., 1.}	99.7531	2.64934	newly 5 weeks spec	14.	0.02	{0., 0.}	99.8571	2.12613	newly 5 weeks cumul	459.	1.06	{1., 1.}	99.7691	2.645	newly 51 weeks cumul	486.	1.06	{1., 1.}	99.7819	2.66982
21	newly 3 weeks spec	38.	0.06	{0., 0.}	99.8421	2.51978	newly 3 weeks cumul	270.	1.03	{1., 1.}	99.6185	2.43631	newly 5 weeks spec	12.	0.	{0., 0.}	100.	2.07918	newly 5 weeks cumul	303.	1.05	{1., 1.}	99.6535	2.46639	newly 52 weeks cumul	316.	1.05	{1., 1.}	99.6677	2.48464
22	newly 3 weeks spec	162.	10.35	{6., 15.}	93.6111	1.22722	newly 3 weeks cumul	806.	51.49	{43., 59.}	93.6117	1.19785	newly 5 weeks spec	58.	3.74	{1., 6.}	93.5517	1.2847	newly 5 weeks cumul	951.	61.37	{53., 69.}	93.5468	1.193	newly 48 weeks cumul	1042.	67.16	{56., 77.}	93.5547	1.19352
23	newly 3 weeks spec	22.	16.66	{11., 22.}	26.	0.144036	newly 3 weeks cumul	142.	87.35	{74., 99.}	38.4859	0.213406	newly 5 weeks spec	3.	5.48	{2., 10.}	96.6667	0.275639	newly 5 weeks cumul	156.	102.03	{87., 115.}	34.5962	0.186933	newly 49 weeks cumul	161.	111.04	{94., 128.}	31.0311	0.164389
24	newly 3 weeks spec	51.	0.08	{0., 0.}	99.8431	2.62757	newly 3 weeks cumul	312.	0.98	{1., 1.}	99.6859	2.54211	newly 5 weeks spec	15.	0.02	{0., 0.}	99.8667	2.15609	newly 5 weeks cumul	360.	1.06	{1., 1.}	99.7056	2.53824	newly 51 weeks cumul	369.	1.06	{1., 1.}	99.7127	2.54896
25	newly 3 weeks spec	160.	4.87	{2., 8.}	96.9563	1.57508	newly 3 weeks cumul	871.	25.1	{16., 33.}	97.1183	1.55809	newly 5 weeks spec	60.	2.23	{1., 4.}	96.2833	1.58024	newly 5 weeks cumul	1024.	30.86	{19., 41.}	96.9863	1.53945	newly 47 weeks cumul	1086.	35.37	{22., 47.}	96.7431	1.50561
26	newly 3 weeks spec	96.	458.96	{436., 484.}	378.083	0.679145	newly 3 weeks cumul	682.	2018.63	{1977., 2050.}	195.987	0.471228	newly 5 weeks spec	33.	233.52	{214., 254.}	607.636	0.848735	newly 5 weeks cumul	784.	2578.52	{2538., 2615.}	228.893	0.517023	newly 45 weeks cumul	878.	3135.98	{3083., 3190.}	257.173	0.552843
27	newly 3 weeks spec	572.	41.25	{32., 50.}	92.7885	1.14849	newly 3 weeks cumul	2438.	176.2	{164., 191.}	92.7728	1.14181	newly 5 weeks spec	284.	22.69	{17., 29.}	92.0106	1.10652	newly 5 weeks cumul	3101.	230.6	{215., 246.}	92.5637	1.12922	newly 45 weeks cumul	3756.	289.37	{274., 304.}	92.2958	1.11363
28	newly 3 weeks spec	8.	15.1	{10., 21.}	89.5	0.260366	newly 3 weeks cumul	54.	126.64	{111., 140.}	134.519	0.368538	newly 5 weeks spec	1.	2.67	{0., 6.}	191.	0.463777	newly 5 weeks cumul	61.	135.68	{120., 151.}	122.426	0.345322	newly 52 weeks cumul	63.	137.68	{121., 154.}	118.54	0.337615
29	newly 3 weeks spec	378.	16.09	{12., 20.}	95.7434	1.3808	newly 3 weeks cumul	1806.	88.03	{79., 98.}	95.1257	1.31377	newly 5 weeks spec	145.	5.82	{3., 9.}	95.9862	1.43569	newly 5 weeks cumul	2196.	103.15	{93., 114.}	95.3028	1.32981	newly 49 weeks cumul	2473.	112.08	{100., 125.}	95.4679	1.34528
30	newly 3 weeks spec	146.	0.	{0., 0.}	100.	3.16435	newly 3 weeks cumul	831.	1.33	{1., 2.}	99.84	2.82651	newly 5 weeks spec	47.	0.	{0., 0.}	100.	2.6721	newly 5 weeks cumul	975.	1.33	{1., 2.}	99.8636	2.89591	newly 52 weeks cumul	1051.	1.33	{1., 2.}	99.8735	2.92851
31	newly 3 weeks spec	292.	197.17	{180., 216.}	32.476	0.171652	newly 3 weeks cumul	1423.	892.45	{869., 920.}	37.2839	0.202732	newly 5 weeks spec	119.	92.81	{80., 105.}	22.0252	0.110435	newly 5 weeks cumul	1719.	1120.93	{1089., 1149.}	34.7917	0.185812	newly 42 weeks cumul	1955.	1324.34	{1283., 1369.}	32.2588	0.169293
32	newly 3 weeks spec	532.	1.51	{0., 3.}	99.7162	2.81893	newly 3 weeks cumul	2380.	6.34	{1., 12.}	99.7336	2.70994	newly 5 weeks spec	254.	0.59	{0., 2.}	99.7677	2.9544	newly 5 weeks cumul	3005.	7.95	{2., 15.}	99.7354	2.70748	newly 48 weeks cumul	3596.	9.38	{2., 18.}	99.7392	2.71068
33	newly 3 weeks spec	65.	0.13	{0., 1.}	99.8	2.6899	newly 3 weeks cumul	390.	0.95	{1., 1.}	99.7564	2.67027	newly 5 weeks spec	28.	0.04	{0., 0.}	99.8571	2.40716	newly 5 weeks cumul	468.	1.05	{1., 1.}	99.7756	2.66343	newly 52 weeks cumul	508.	1.06	{1., 1.}	99.7913	2.68905
34	newly 3 weeks spec	107.	63.12	{55., 73.}	41.0093	0.232335	newly 3 weeks cumul	548.	320.45	{306., 335.}	41.5237	0.233288	newly 5 weeks spec	35.	23.86	{18., 29.}	31.8286	0.174746	newly 5 weeks cumul	648.	382.06	{370., 397.}	41.0401	0.229609	newly 48 weeks cumul	692.	421.29	{410., 434.}	39.1199	0.215673
35	newly 3 weeks spec	10.	0.09	{0., 0.}	99.1	1.91699	newly 3 weeks cumul	81.	1.24	{1., 2.}	98.4691	1.85272	newly 5 weeks spec	0.1	0.01	{0., 0.}	108.	0.01	newly 5 weeks cumul	82.	1.27	{1., 2.}	98.4512	1.83503	newly 52 weeks cumul	82.	1.27	{1., 2.}	98.4512	1.83503
36	newly 3 weeks spec	587.	1.4	{0., 3.}	99.7615	2.94187	newly 3 weeks cumul	2638.	7.95	{2., 14.}	99.6986	2.64387	newly 5 weeks spec	275.	0.45	{0., 2.}	99.8364	3.10617	newly 5 weeks cumul	3306.	9.32	{2., 18.}	99.7181	2.6634	newly 48 weeks cumul	3908.	10.38	{2., 19.}	99.7344	2.68105
37	newly 3 weeks spec	138.	0.18	{0., 1.}	99.8696	2.95988	newly 3 weeks cumul	721.	0.78	{0., 1.}	99.8918	3.0989	newly 5 weeks spec	53.	0.05	{0., 0.}	99.9057	2.67428	newly 5 weeks cumul	854.	0.96	{1., 1.}	99.8876	2.99942	newly 49 weeks cumul	922.	1.05	{1., 1.}	99.8861	2.94968
38	newly 3 weeks spec	103.	88.58	{78., 99.}	14.466	0.0694452	newly 3 weeks cumul	598.	434.74	{416., 451.}	27.301	0.138678	newly 5 weeks spec	29.	38.47	{29., 47.}	33.2759	0.118922	newly 5 weeks cumul	693.	530.78	{512., 550.}	23.4084	0.115965	newly 46 weeks cumul	752.	599.56	{578., 619.}	20.2713	0.0985376
39	newly 3 weeks spec	188.	51.11	{42., 60.}	72.8138	0.569318	newly 3 weeks cumul	946.	286.52	{272., 300.}	69.7125	0.518994	newly 5 weeks spec	61.	18.18	{13., 22.}	70.1967	0.537228	newly 5 weeks cumul	1112.	333.84	{320., 347.}	69.9784	0.522774	newly 48 weeks cumul	1224.	358.87	{344., 372.}	70.6806	0.533033
40	newly 3 weeks spec	395.	25.59	{19., 31.}	93.5215	1.19597	newly 3 weeks cumul	1868.	138.76	{126., 148.}	92.5717	1.12992	newly 5 weeks spec	218.	10.07	{6., 14.}	95.3807	1.35776	newly 5 weeks cumul	2391.	165.39	{154., 178.}	93.0828	1.16068	newly 49 weeks cumul	2892.	180.59	{168., 192.}	93.7555	1.20508
41	newly 3 weeks spec	12.	0.06	{0., 0.}	99.5	2.01918	newly 3 weeks cumul	121.	1.32	{1., 2.}	98.9091	2.00793	newly 5 weeks spec	2.	0.	{0., 0.}	100.	1.30103	newly 5 weeks cumul	135.	1.35	{1., 2.}	99.	2.03372	newly 52 weeks cumul	137.	1.35	{1., 2.}	99.0146	2.04011
42	newly 3 weeks spec	675.	635.61	{607., 664.}	5.93926	0.0268486	newly 3 weeks cumul	2966.	2781.9	{2738., 2823.}	6.20701	0.0278595	newly 5 weeks spec	340.	316.75	{294., 341.}	7.32059	0.0335222	newly 5 weeks cumul	3794.	3548.17	{3512., 3584.}	6.47944	0.0291057	newly 43 weeks cumul	4690.	4313.94	{4279., 4346.}	8.01834	0.0363073
43	newly 3 weeks spec	545.	102.34	{90., 114.}	81.222	0.728469	newly 3 weeks cumul	2315.	488.37	{469., 507.}	78.9041	0.676021	newly 5 weeks spec	245.	46.07	{37., 55.}	81.1959	0.731121	newly 5 weeks cumul	2918.	602.94	{583., 623.}	79.3372	0.68495	newly 48 weeks cumul	3370.	691.82	{668., 712.}	79.4712	0.687759
44	newly 3 weeks spec	215.	0.77	{0., 2.}	99.6419	2.72445	newly 3 weeks cumul	1211.	4.3	{1., 8.}	99.6449	2.54685	newly 5 weeks spec	93.	0.1	{0., 0.}	99.8925	2.86848	newly 5 weeks cumul	1475.	4.73	{1., 8.}	99.6793	2.57945	newly 48 weeks cumul	1690.	4.9	{1., 8.}	99.7101	2.62508
45	newly 3 weeks spec	201.	0.34	{0., 1.}	99.8308	3.01337	newly 3 weeks cumul	1070.	4.15	{1., 7.}	99.6121	2.48945	newly 5 weeks spec	90.	0.04	{0., 0.}	99.9556	2.91424	newly 5 weeks cumul	1275.	4.24	{1., 7.}	99.6675	2.55706	newly 50 weeks cumul	1419.	4.24	{1., 7.}	99.7012	2.60353
46	newly 3 weeks spec	138.	0.04	{0., 0.}	99.971	3.10687	newly 3 weeks cumul	722.	1.07	{1., 1.}	99.8518	2.85144	newly 5 weeks spec	50.	0.	{0., 0.}	100.	2.69897	newly 5 weeks cumul	861.	1.1	{1., 1.}	99.8722	2.9049	newly 52 weeks cumul	942.	1.1	{1., 1.}	99.8832	2.94395
47	newly 3 weeks spec	224.	41.47	{33., 49.}	81.4866	0.738149	newly 3 weeks cumul	1089.	212.33	{201., 224.}	80.5023	0.710462	newly 5 weeks spec	77.	15.92	{12., 20.}	79.3247	0.696991	newly 5 weeks cumul	1298.	253.78	{240., 268.}	80.4484	0.709203	newly 47 weeks cumul	1446.	280.87	{265., 297.}	80.5761	0.712076
48	newly 3 weeks spec	86.	0.64	{0., 2.}	99.2558	2.41633	newly 3 weeks cumul	493.	4.16	{1., 8.}	99.1562	2.15741	newly 5 weeks spec	18.	0.1	{0., 0.}	99.4444	2.15527	newly 5 weeks cumul	561.	4.51	{1., 8.}	99.1961	2.1803	newly 50 weeks cumul	581.	4.66	{1., 9.}	99.1979	2.18544
49	newly 3 weeks spec	117.	290.1	{268., 312.}	147.949	0.393716	newly 3 weeks cumul	638.	1477.17	{1448., 1502.}	131.531	0.364571	newly 5 weeks spec	40.	114.37	{98., 129.}	185.925	0.454122	newly 5 weeks cumul	753.	1775.56	{1754., 1799.}	135.798	0.372519	newly 48 weeks cumul	828.	1974.55	{1950., 1994.}	138.472	0.377423
50	newly 3 weeks spec	261.	0.92	{0., 2.}	99.6475	2.74906	newly 3 weeks cumul	1311.	4.95	{2., 9.}	99.6224	2.51025	newly 5 weeks spec	104.	0.43	{0., 1.}	99.5865	2.65818	newly 5 weeks cumul	1579.	5.91	{2., 11.}	99.6257	2.51534	newly 49 weeks cumul	1754.	6.48	{2., 12.}	99.6306	2.52495
